# Effect of hydration on the anatomical form of human dry skulls

**DOI:** 10.1038/s41598-022-27042-9

**Published:** 2022-12-29

**Authors:** Konstantinos Dritsas, Jannis Probst, Yijin Ren, Carlalberta Verna, Christos Katsaros, Demetrios Halazonetis, Nikolaos Gkantidis

**Affiliations:** 1 Department of Orthodontics and Dentofacial Orthopedics, School of Dental Medicine, University of Bern, 3010 Bern, Switzerland; 2 Department of Orthodontics, W.J. Kolff Institute, University Medical Center Groningen, University of Groningen, 9700RB Groningen, The Netherlands; 3 Department of Pediatric Oral Health and Orthodontics, UZB-University Center for Dental Medicine, University of Basel, 4058 Basel, Switzerland; 4 Department of Orthodontics, School of Dentistry, National and Kapodistrian University of Athens, 11527 Athens, Greece

**Keywords:** Medical research, Diagnosis, Medical imaging, Bone imaging, Radiography, Three-dimensional imaging

## Abstract

In radiology research soft tissues are often simulated on bone specimens using liquid materials such as water, or gel-like materials, such as ballistic gel. This study aimed to test the effect of hydration on the anatomical form of dry craniofacial bone specimens. Sixteen human dry skulls and 16 mandibles were scanned with an industrial scanner in dry conditions and after water embedding. Ten skulls were also embedded for different time periods (5 or 15 min). The subsequent 3D surface models were best-fit superimposed and compared by calculating mean absolute distances between them at various measurement areas. There was a significant, primarily enlargement effect of hydration on the anatomical form of dry skeletal specimens as detected after water embedding for a short time period. The effect was smaller in dry skulls (median 0.20 mm, IQR 0.17 mm) and larger in mandibles (median 0.56 mm, IQR 0.57 mm). The effect of different water embedding times was negligible. Based on the present findings, we suggest to shortly hydrate the skeletal specimens prior to reference model acquisition so that they are comparable to hydrated specimens when liquid materials are used as soft-tissue simulants for various radiologic research purposes.

## Introduction

In radiology, research strives to improve image quality whilst keeping the patient exposure to radiation as low as reasonably achievable (ALARA principle)^1^. Human subjects are rarely used for research purposes, due to ethical considerations, especially when repeated exposures are required to test various imaging protocols. For this reason, dry human cadaveric specimens are often used instead in ex vivo experimentation to develop and validate X-ray imaging techniques^2,3^. A limitation of this approach stems from the fact that no soft tissues are present, which affects the resulting image, and thus, the applicability of outcomes in actual clinical conditions^4^. To overcome this limitation, soft tissues can be simulated using liquid materials such as water, wax sheets, or gel-like materials, such as ballistic gel^5,6^.

The accuracy of radiographic imaging techniques is often tested through the comparison of a reference three-dimensional (3D) digital model with a model acquired through the radiographic method in question. The reference model depicts the object as closely to reality as possible, and in modern research, it is often acquired through direct surface scanning of the object of interest using accurate structured-light scanners^3,7^. The latter option offers the advantage of being free from segmentation error, which is always present when segmenting 3D surface models from radiographic images (computed tomographies or cone beam computed tomographies)^7,8^. Furthermore, modern surface scanners offer very high resolution images that cannot be usually obtained through regular radiographic imaging techniques^9–11^.

Due to the above considerations, in radiological research a common strategy in diagnostic accuracy studies is the comparison of direct surface scans of dry skeletal structures, with radiographic images of the same objects, which are though embedded in liquid materials for soft-tissue simulation^2,3,5–7^. However, potential water absorption during the dry bone hydration process might affect the anatomical form of the tested bony specimens (e.g., slight changes in size or shape), which might impact the accuracy outcomes^12^. Such possibility has not yet been investigated and might be an additional source of error when comparing digital models directly derived from dry skeletal surfaces to models derived from CT or CBCT imaging, where soft-tissue simulation through liquid materials was implemented.

Thus, the aim of this study was to test the effect of hydration on the anatomical form of dry craniofacial bone specimens, through the evaluation of 3D digital surface models acquired before and after hydration with a high accuracy hand-held surface scanner. Human dry skulls and mandibles were used for this purpose. The effect of hydration time on dry skull form was also tested as a secondary outcome.

## Materials and methods

### Specimens

The sample consisted of 16 dry skulls and 16 mandibles from human cadavers, that were acquired from the Municipal cemetery of Serres, Greece following the necessary authorization provided by the local authorities (Municipality of Serres, Greece, Protocol Number: 4044/12.07.2018). All handling of human tissues was compliant with the relevant local legislation. The specimens belonged to humans that were deceased between 8 and 12 years prior to the implementation of the present study. At the time of acquisition, there were no claims from any relatives and the specimens were buried altogether according to the cemetery protocol. At this point, the identity of the specimens was not known, and therefore, no informed consent was sought. The upper part of the cranium was removed, so that the anterior cranial base surface could be accessed from the sensors of the surface scanners.

### Ethics declaration

The study was conducted in accordance with the Declaration of Helsinki, and approved by the Institutional Review Board of National and Kapodistrian University of Athens (Protocol Number: 335/02.05.2017).

### Data acquisition

All 32 bone specimens were initially scanned in dry conditions with the structured-light 3D surface scanner Artec Space Spider (Artec3D, Luxembourg, Software: Artec Studio 12, Version 12.1.6.16). Throughout the experiment, the specimens were kept in a room in Athens, with 22–25 °C temperature, natural as well as artificial ambient light, and good natural ventilation. Prior to scanning the scanner was calibrated according to the manufacturer’s instructions. Regarding the skulls, the complete outer facial surface and the inner anterior cranial base were the primary target areas, although the complete image of the skull was acquired. In the mandibles, the entire surface comprised the primary target area. During the scanning procedure, multiple overlapping partial scans were acquired, which were later processed as described in the next section to obtain the final 3D surface models used in the study.

In a second step, all skulls were embedded in tap water for 15 min and all mandibles for 10 min and were afterwards rescanned, shortly after their removal from the water. The water was in room temperature (22–25 °C) and had a pH of approximately 7.5. To reduce light scattering created by water droplets on the bony surfaces, and thus, reduce potential artifacts, all specimens were gently patted with tissue paper before the scan.

To test the effect of embedding time on the outcomes, 10 dry skulls were embedded in water for 5 min and scanned as described above. Immediately after scan, and while still being hydrated, the same skulls were embedded in water for additional 10 min (total embedding time 15 min) and were rescanned with the same process. This part of the experiment was performed a few days apart from the previous experimentation, so that the specimens had occupied their original dry condition prior to the implementation of this part.

One trained operator (NG) acquired all the scans, using the same methodology, which was defined based on the manufacturer’s instructions and following pilot testing. Every scan was performed within a one-month period in a room with average room temperature (22–25 °C) and natural^10,11^, as well as artificial light. A more detailed description of the image acquisition process and an assessment of the associated scanner error have been published previously^11^. The median scanner error amounted to approximately 28 μm in dry conditions, whereas it was slightly higher at wet conditions, at 40 μm^9^.

### Data post-processing (3D model generation)

Another trained operator (JP) processed the raw scan data obtained for each specimen in the Artec Studio 16 software (Version 16.0.5.114), to combine the partial scans into a single complete 3D model of each skull and mandible. The required data processing for the final surface model included various steps which were controlled manually^10,11^. Imprecise single images, visible artifacts and hard tissues away from the area of interest of each partial scan were manually deleted, targeting a maximum error of 0.3 among all scans. The initial alignment of the partial scans was then performed either solely through the auto-alignment function or through an initial rough approximation using homologous landmarks, followed by the auto-alignment function. The final registration of the scans consisted of a rough serial registration, using only geometry, and a global registration at a key frame ratio of 0.3. Remaining outliers were removed using the following settings: 3D-noise level 3, 3D resolution 0.3 mm and the final complete 3D models were generated by sharp fusion of all partial scans at a resolution of 0.3 mm, without filling holes. The resulting 3D models were exported as standard tessellation language (STL) files for further analysis. A more detailed description of the post-processing 3D model generation process as well as an error assessment of this process has been published previously. The 3D model generation error was negligible (5–10 μm)^11^.

### Hydration effect

**Figure 1:**
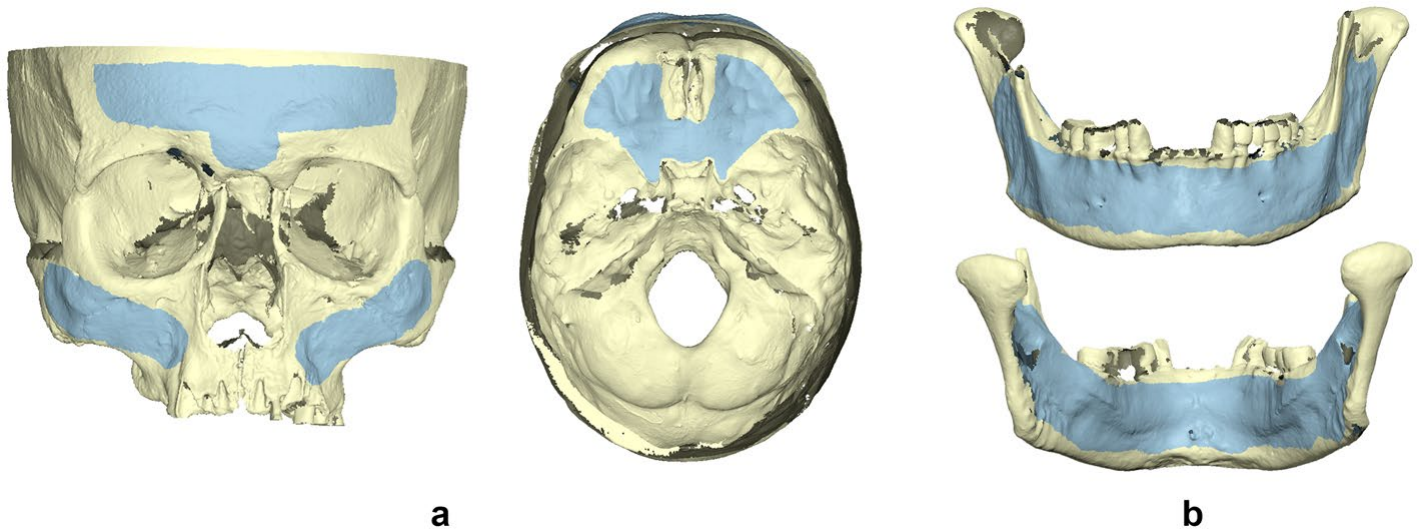
Superimposition reference areas used in the (**a**) skulls and (**b**) mandibles (top: buccal aspect, bottom: lingual aspect) to register corresponding 3D surface models, depicted in light blue color. *Adapted from: Probst *et al*. Diagnostics 2022, 12, 2251. *https://doi.org/10.3390/diagnostics12092251^11^.

Each 3D surface model that was acquired in dry conditions was superimposed to its corresponding hydrated model through water embedding, using Viewbox 4 software (Version 4.1.0.12, dHAL Software, Kifissia, Greece) and the implementation of an iterative closest point algorithm (ICP)^13^, with the following settings: 100% estimated overlap, exact nearest neighbor search, matching point to plane, 100% point sampling, and 50 iterations. The selected superimposition reference areas for each skull or mandible are presented in Fig. 1 and have been thoroughly described previously^11^.

**Figure 2:**
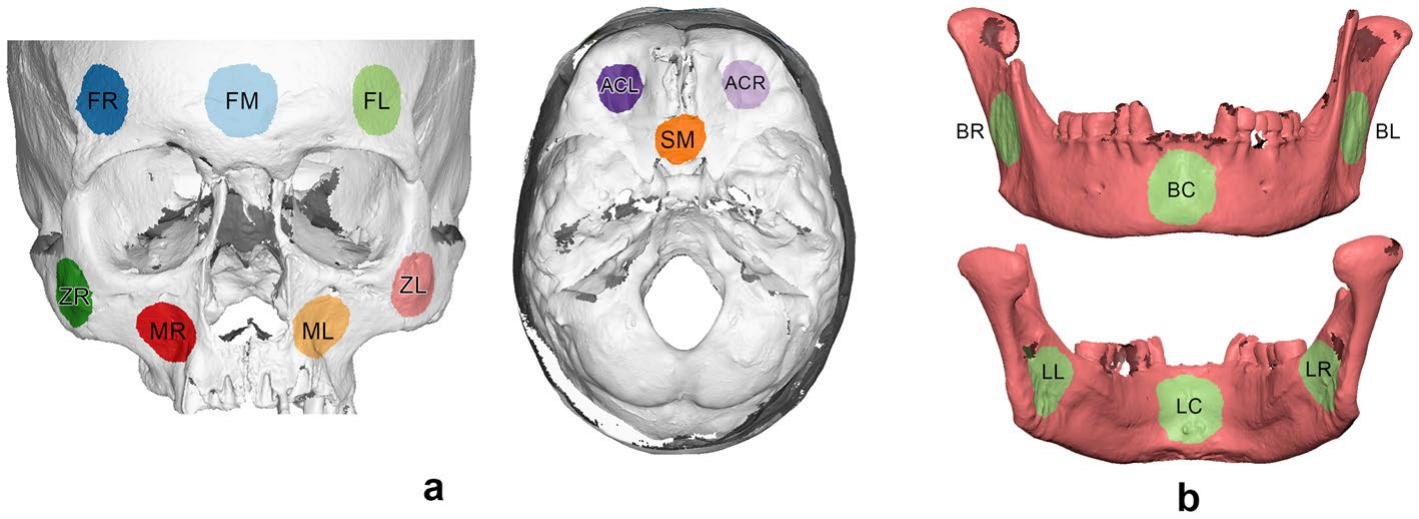
Measurement areas used to assess differences between corresponding 3D surface models shown as colored round surfaces. (**a**) Skull. Forehead left (FL), right (FR) and middle (FM), maxilla left (ML) and right (MR), zygomatic bone left (ZL) and right (ZR), anterior cranial base left (ACL) and right (ACR), sphenoid bone middle (SM). (**b**) Mandible. Upper image: Buccal left (BL), right (BR) and center (BC). Lower image: Lingual left (LL), right (LR) and center (LC). *Adapted from: Probst *et al*. Diagnostics 2022, 12, 2251. *https://doi.org/10.3390/diagnostics12092251^11^.

According to a previously published protocol^11^, ten measurement sites were defined in each skull by selecting a circular area of 10,000 triangles in each site. Six measurement areas were defined in each mandible in a similar way. The location of the measurement areas is shown in Fig. 2 and has been thoroughly described previously^11^.

Changes in the anatomical form of each specimen were quantified by measuring the mean absolute distance (MAD) between each pair of best-fit superimposed scans of the same specimen in wet and dry conditions, at each measurement area. Zero MAD would suggest perfect congruence of the two models, and thus no effect of hydration; the effect would increase in magnitude proportionally to a MAD increase. Differences in the anatomical form of the entire bone specimens in dry and wet conditions were also visualised through colour-coded distance maps between corresponding best-fit superimposed models. Per pair of models, the dry specimen served always as the reference model and the hydrated specimen was approximated to it. In terms of interpretation, when a hydrated surface was positioned closer to the viewer it appears with positive values (i.e. red colours), whereas when it appears further from the viewer (the dry surface appears closer) it receives negative values (i.e. blue colours). The white/grey areas that do not have a corresponding surface on both superimposed models. Thus, these are not measured by the software. The effect of different water embedding times in the anatomical form of the skulls was tested in a similar manner.

### Statistical analysis

The statistical analysis utilized the IBM SPSS statistics for Windows (Version 28.0. Armonk, NY: IBM Corp). Shapiro–Wilk and Kolmogorov–Smirnov tests on the raw data did not consistently detect normal distributions. Therefore, non-parametric statistics were applied, and the differences were expressed using median differences and their interquartile range values (IQR).

The differences between corresponding 3D surface models scanned in wet and dry conditions are shown with box plots for each measurement area. Differences among measurement sites were tested through Kruskal–Wallis’s test, followed by Dunn’s test for pairwise comparisons, if statistically significant differences were found. Significance values were adjusted by the Bonferroni correction for multiple tests. The statistical significance level was defined at 0.05.

## Results

### Dry versus hydrated skulls

**Figure 3:**
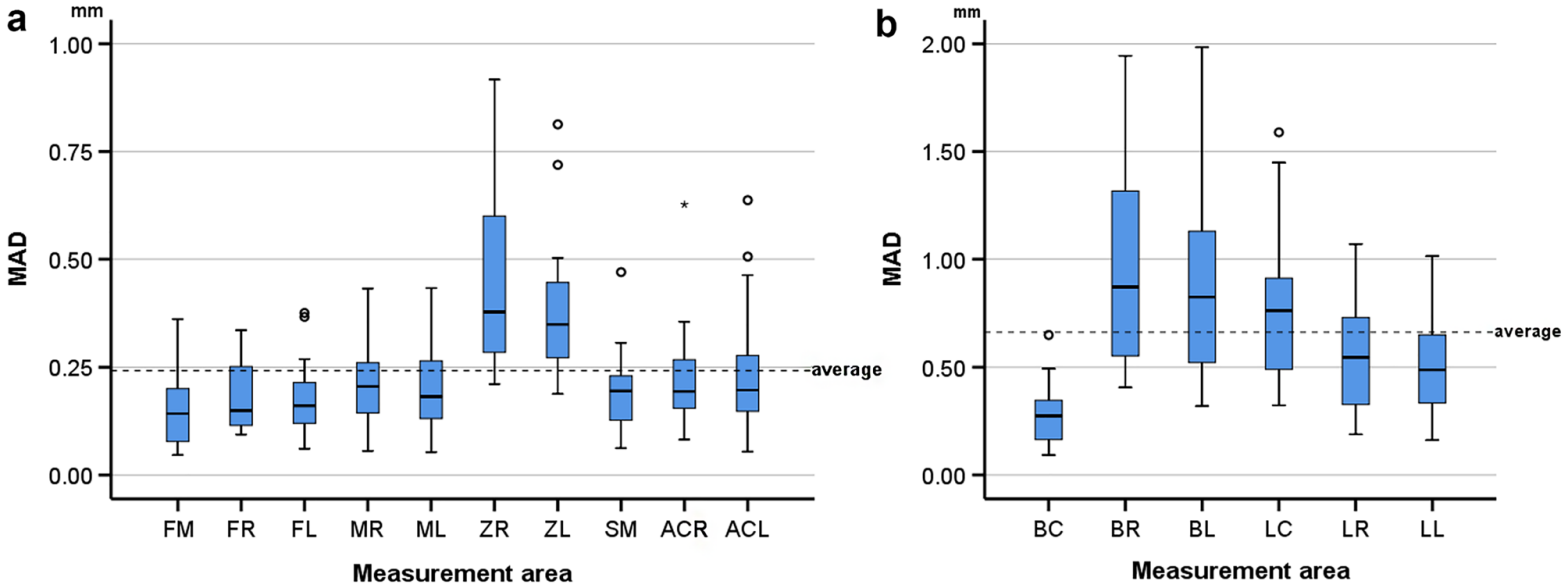
Box plots showing the MAD between the wet and dry models at each measurement area in (**a**) the skulls and (**b**) the mandibles. MAD: mean absolute distance. Skull: forehead middle (FM), right (FR) and left (FL), maxilla right (MR) and left (ML), zygomatic bone right (ZR) and left (ZL), sphenoid bone middle (SM) and anterior cranial base right (ACR) and left (ACL). Mandible: buccal center (BC), right (BR) and left (BL) and lingual center (LC), right (LR) and left (LL). Outliers are shown as black circles (further from the median more than 1.5 times the IQR) or asterisks in more extreme cases (further from the median more than 3 times the IQR).

**Figure 4:**
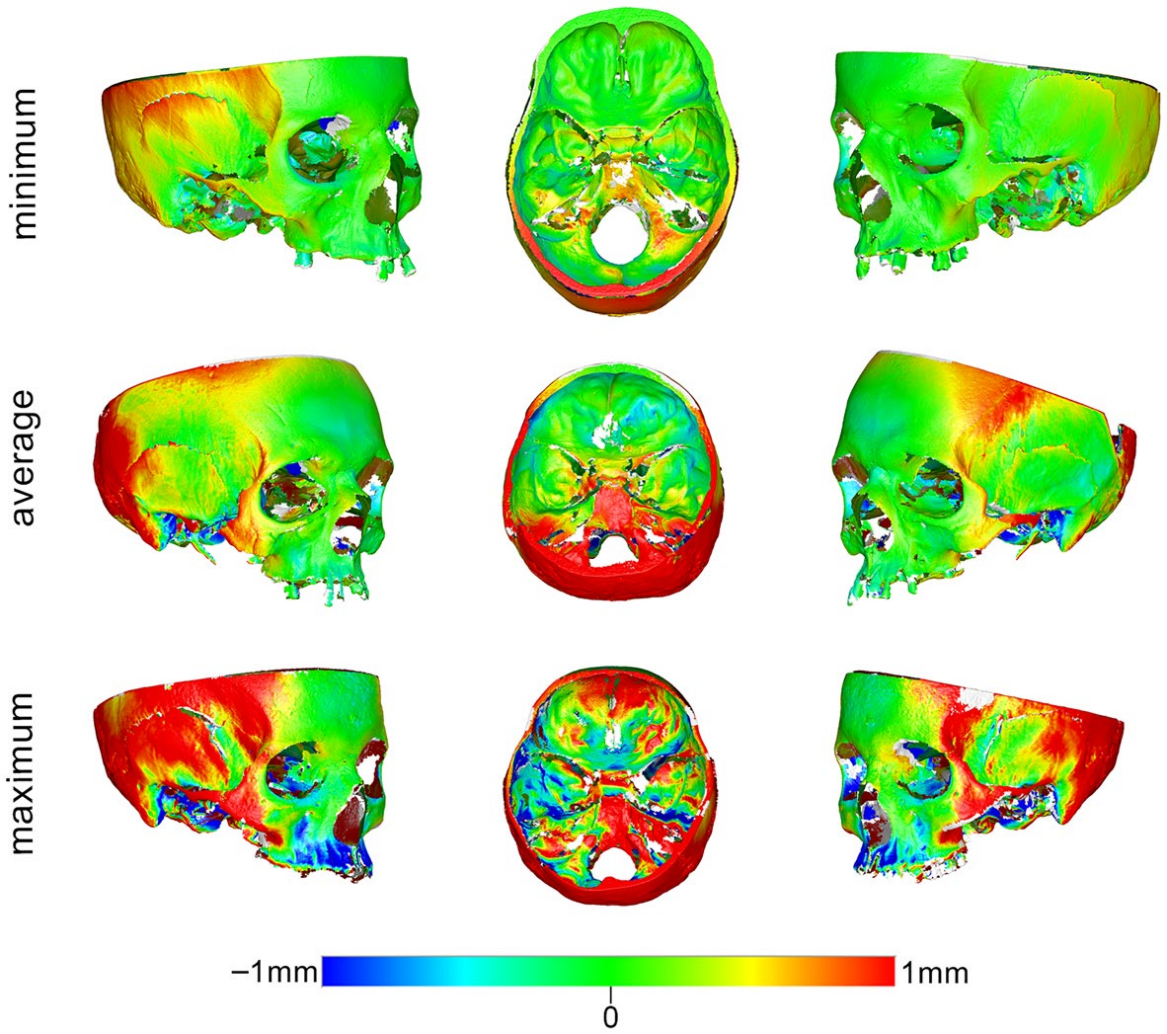
Color coded distance maps of skulls showing the difference between dry (reference) and wet models, depicted through selected models that corresponded to the minimum, average and maximum error detected in the pre-specified measurement areas. Zero distance between best-fit approximated repeated scans indicates perfect reproducibility.

The median difference (MAD) between skull models before and after hydration was 0.205 mm (IQR 0.173; range 0.046, 0.917). The measured difference was found to be affected by the location of the measurement site (Kruskal–Wallis’ test: *p* < 0.001). Pairwise comparisons revealed significant differences of the two zygomatic areas (ZL, ZR) from almost all other areas (FM, FL, FR, ML, SM) and of the right zygomatic area (ZR) with the right maxillary area (MR) and the right anterior cranial base area (ACR) (*p* < 0.05, after Bonferroni adjustment). Among all measurement sites, the zygomatic areas exhibited the strongest effect, as revealed by visual inspection of the box plots in Fig. 3a. A thorough assessment of the differences of the tested specimens through colour coded distance maps was indicative of an enlargement of the hydrated models. Three models, representative of the tested sample, are shown in Fig. 4. The findings are in accordance with the quantitative outcomes reported above. Relevant assessment of all models is available in the Supplementary Fig. S1.

### Dry versus hydrated mandibles

**Figure 5:**
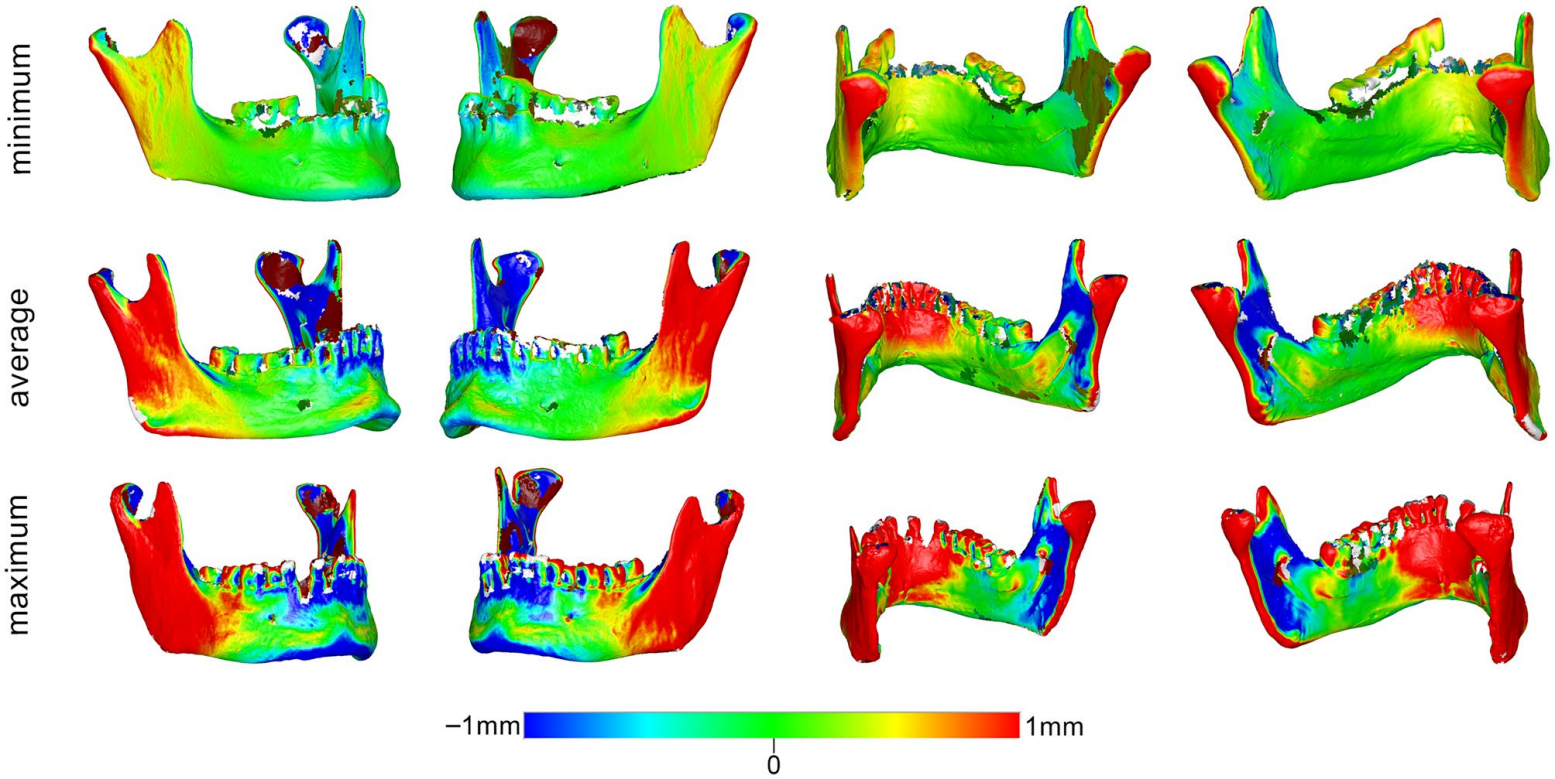
Color coded distance maps of mandibles showing the difference between dry (reference) and wet models, depicted through models that corresponded to the minimum, average and maximum error detected in the pre-specified measurement areas. Zero distance between best-fit approximated repeated scans indicates perfect reproducibility.

The median difference (MAD) between the mandibles before and after water embedding was 0.562 mm (IQR 0.573; range 0.092, 1.984). The measured differences were affected by the location of the measurement site (Kruskal–Wallis’ test: *p* < 0.001). Pairwise comparisons revealed significant differences between the buccal chin area (BC) with the lingual chin area (LC) and both lateral sides of the rami (BL, BR) (*p* < 0.05, after Bonferroni adjustment). Among all measurement sites, the lingual area of the chin (LC) and the lateral sides of the rami (BR, BL) exhibited the greatest differences, as revealed by visual inspection of the box plots in Fig. 3b. A thorough assessment of the differences of the tested specimens through colour coded distance maps was indicative of an enlargement of the hydrated models, as well as a bilateral expansion combined with a slight flattening of the mandibular curvature. Three models, representative of the tested sample, are shown in Fig. 5. The findings are in accordance with the quantitative outcomes reported above. Relevant assessment of all models is available in the Supplementary Fig. S2.

### Effect of embedding time on skull form

**Figure 6:**
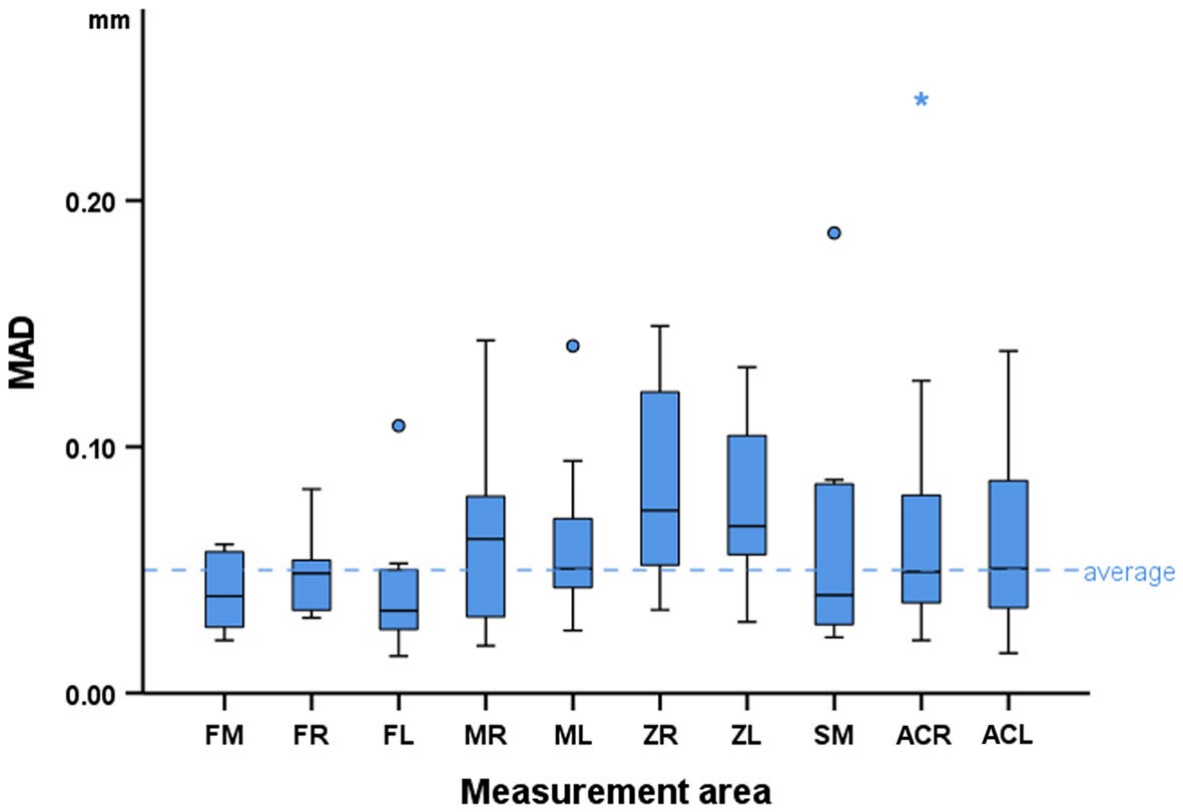
Box plots showing the MAD between dry skull models embedded in water for 5 and 15 min, at each measurement area. MAD: mean absolute distance. Skull: forehead left (FL), right (FR), and middle (FM), maxilla left (ML) and right (MR), zygomatic bone left (ZL) and right (ZR), anterior cranial base left (ACL) and right (ACR) and sphenoid bone middle (SM). Mandible: buccal left (BL), right (BR), and center (BC) and lingual left (LL), right (LR) and center (LC). Outliers are shown as blue dots (further from the median more than 1.5 times the IQR) or stars in more extreme cases (further from the median more than 3 times the IQR).

**Figure 7:**
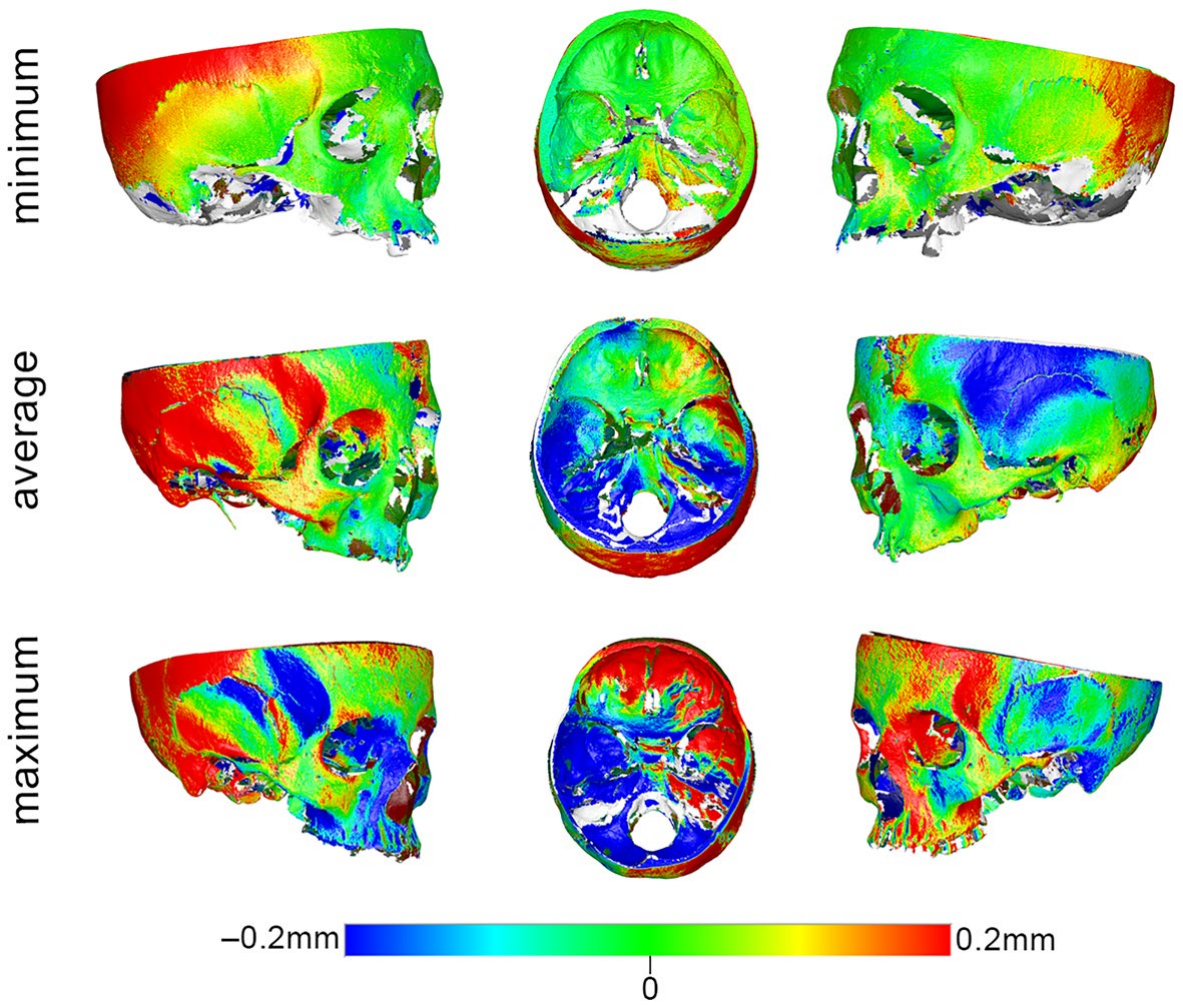
Color coded distance maps showing the difference between skulls embedded in water for different time periods (5 min set as reference and 15 min), depicted through selected models that corresponded to the minimum, average and maximum error detected in the pre-specified measurement areas. Zero distance between best-fit approximated repeated scans indicates zero effect of the embedding time.

The median overall difference between best-fit superimposed corresponding skulls that were embedded in water for different time periods (5 and 15 min) was of limited magnitude (median 0.051 mm; IQR 0.047; range 0.015, 0.241). The location of the measurement area was found to mediate this effect (Friedman’s test: p = 0.008). Pairwise comparisons revealed significant differences only between the following pairs: ZR versus FM and ZR versus FL (Dunn’s test: *p* = 0.023 and *p* = 0.040, respectively) (Fig. 6). Differences at three entire models representative of the tested sample are available in the colour coded distance maps shown in Fig. 7. All models are shown in the Supplementary Fig. S3.

## Discussion

The median difference between the models scanned in dry and hydrated conditions was 0.20 mm for the skulls and 0.56 mm for the mandibles; a difference noticeably bigger than the scanner error, which averages at less than 0.05 mm for both structures, according to a previous publication^11^. In individual cases, the hydration effect approached 1 mm in the skulls and 2 mm in the mandibles. Thus, the hydration of the skeletal specimens was found to significantly affect their form, which has direct implications for the usage of dry skeletal models as true reference models in ex vivo radiology research. In such studies, it is common practice to scan the skeletal specimens in dry conditions and obtain the true reference and then embed them to liquid materials for soft-tissue simulation during radiographic image acquisition. However, the present findings indicate that the two models are not expected to be identical due to the different hydration conditions of the skeletal specimens. Based on the present findings, we suggest hydrating the skeletal specimens prior to reference model acquisition, e.g. through 3D surface scanning, so that they are directly comparable to the hydrated test specimens that are often subjected to radiographic images.

In the skulls, there were more pronounced differences at the temporal bones and the posterior areas, which often exceeded 1 mm, but this is attributed to rotational effects due to the fact that these areas were located further from the superimposition reference areas. The corresponding models were superimposed on areas residing mostly at the front of the skull. Thus, following the best fit approximation, small model rotations around the superimposition reference areas led to greater distances between corresponding models as the distance from the rotation centre, located somewhere within the reference areas, increased; a notion that has been well established in the literature^9,11,14–17^. If we consider that all measurement areas of the skull are located on or near the superimposition references areas, it is a valid argument that the zygomatic areas showed higher differences between dry and hydrated skulls. A possible explanation for this could be that the zygomatic arches are located at the corners of the skull and are connected only at their ends to the rest of the skull. Thus, because the zygomatic areas are not as solidly connected to the rest of skull as the other skeletal structures, which have continuous connections to each other, they are less robust. Even in the two zygomatic areas, the median differences between dry and hydrated skulls where below 0.5 mm and never exceeded 1 mm in any individual case.

On the other hand, in the mandibles, where the superimposition reference area was evenly distributed over the entire mandibular surface, unbiased comparisons were possible between any measurement area. There, notable differences were evident at the ramus and the central lingual area of the mandibles, as revealed by the visual inspection of the respective colour maps, which even approximated 2 mm in certain individual cases. It seems that the distal part of the mandibles (ramus and condyle) expanded bilaterally, and the central mandibular area shifted inwards, whereas the area in-between remained unchanged. The shift in shape is consistent with a flattening of the overall mandibular arch (Supplementary Fig. S4) and was apparent in all mandibular specimens to various extents. A similar effect, but less pronounced, was also observed in 4 out of the 16 skull models, with the zygomatic bones expanding bilaterally and the inner part of the forehead shifting inwards. A possible explanation of the diverse hydration effects between the skulls and the mandibles could be that the skull has a closed round shape, in contrast to the open shape of the mandible, and that the skull’s inner bony structures are interconnected and might provide some morphological robustness.

Although it was clearly apparent that the mandibles exhibited greater changes due to hydration than the skulls, a statistical test was not performed to directly assess this finding. The reason was the substantial differences in the number and extent of the measurement sites of the skulls compared to these of the mandibles; differences that might have introduced bias if a direct comparison was made. Moreover, unpaired comparisons between measurement areas were performed, aiming to test for the overall effect in a group of specimens and not weighing on within specimen effects (that would be the paired case). Within specimen effects can be still assessed through the colour-coded distance maps provided for selected specimens in Figs. 4, 5, and 7 and for each single specimen in the supplementary file. These figures show the respective effects in detail, and there, it is evident that there is a within specimen effect, which however is beyond the scope of the study to be further investigated.

A possible correlation between the initial dry specimen volume and the overall hydration effect was investigated in the mandibles in an exploratory manner. For consistency, the tooth surfaces were manually removed from the alveolar process and any remaining holes were closed in a semi-automated way using the Viewbox software’s hole-filling algorithm. This allowed the creation of a watertight model, which is required for volume calculation. The latter was then correlated with the detected MAD between the dry and wet specimens. Both linear regression (Supplementary Fig. S5) and bivariate correlation were not statistically significant (Spearman’s test: *p* = 0.863) indicating the absence of mediation of outcomes by the original volume of the specimens. No such testing was performed in the skulls, because the hole-filling process, required for volume calculation, would be highly error-prone due to the complex skull anatomy. The mandibles have a simpler shape, and thus, less confounding by the hole filling process is expected.

The overall 3D model generation error due to the image stitching process required to unify the partial scans to a complete surface model of the skeletal specimens was tested previously in both dry and wet conditions and was found very low (median between 0.005 and 0.010 mm and it never exceeded 0.025 mm)^11^. The scanner precision, which includes the 3D model generation error, was also low, detected at a median of 0.031 mm for dry and 0.040 mm for wet skeletal surfaces. When considering these error levels and the magnitude of the different embedding time (5 and 15 min) on the anatomical form of the dry skulls (approximately 0.050 mm) we can conclude that the effect of water embedding time on the skull form is negligible.

The specific time where the skeletal specimen is embedded to the soft-tissue simulant prior to radiographic image acquisition is usually not reported in relevant studies^6,18,19^. The main embedding period used in the present study was 10 min for the mandibles and 15 min for the skulls to allow for effective hydration of the skeletal specimens and efficient handling to perform the required scans in reasonable time, whereas avoiding permanent damage that could have been caused by an extended hydration period. Moreover, we considered that this time might be representative of the time where a specimen would remain in the simulant prior to radiographic image acquisition during actual experimentation with various skeletal specimens. We arbitrarily chose a slightly longer embedding time for the skulls, since these are bigger structures, and thus, they might require more time to be hydrated. In any case, the different embedding times tested for the skulls did not result in any significant effects on their morphological configuration.

To our knowledge, this is the first study in the literature that investigated the effect of hydration (water absorption) on the anatomical form of dry skeletal specimens. Our findings have important implications, as water is extensively used as soft tissue simulant, because of its ease of use, low cost, and availability, in both its liquid and iced form^5,6,19,20^. Moreover, other commonly used materials, such as ballistic gelatin^21^, might also have a hydration effect due to high amount of water that they usually contain (approximately 80–90%). The water absorption effect proved to be an additional source of error in studies which utilize direct surface scans, or any CT or CBCT data acquired in dry conditions, as gold standard reference models and compare them to data derived from specimens with simulated soft tissues, using liquid substances. For this reason, it should be considered in such studies to acquire the gold standard references either after hydration, e.g. through a short water embedding session, or to use soft tissue simulation materials that do not contain high amounts of water, such as acrylic or wax^2,3,6^. A shortcoming of the latter approach might be the damage of the specimens during the placement or the removal of the soft-tissue simulant, whereas water embedding for long periods might also cause permanent damage.

A limitation of the present study was that it only tested water as a soft tissue simulant material. The extent to which other liquid or hydrogel materials, that also contain water, can hydrate the dry specimens, and affect their anatomical form remains to be investigated. Another limitation regards the duration in which the specimens were embedded in water. Based on the present findings a 5 min embedding does not differ from a 15 min embedding in terms of effects on the anatomical form of the skeletal specimen. However, it remains unknown if shorter or longer embedding times would have different effects.

## Conclusions

There was a significant effect of hydration on the anatomical form of dry skeletal specimens as detected after embedding them in water for a short period. The effect was smaller in skulls and larger in mandibles and has important implications for studies that use liquid soft tissue simulants and compare the radiographic outcomes to the dry models. The effect of different water embedding times (5 versus 15 min) was negligible. Based on the present findings, we suggest to shortly hydrate the skeletal specimens prior to surface scanning, if water is used as a soft-tissue simulant in research projects, where direct 3D surface scans are compared to radiographically derived models of hydrated specimens.

## Supplementary Information

Supplementary Figures.

## Data Availability

The data presented in this study are available on request from the corresponding author.
