# Supplementary material for: Effect of hydration on the anatomical form of human dry skulls
Source: Sci Rep. 2022 Dec 29;12:22549. doi: 10.1038/s41598-022-27042-9 (PMC9800411; doi:10.1038/s41598-022-27042-9)
Supplement: Supplementary file 1 — Supplementary Figures. [file 41598_2022_27042_MOESM1_ESM.docx]

Effect of hydration on the anatomical form of human dry skulls

Konstantinos Dritsas, Jannis Probst, Yijin Ren, Carlalberta Verna, Christos Katsaros, Demetrios Halazonetis, Nikolaos Gkantidis


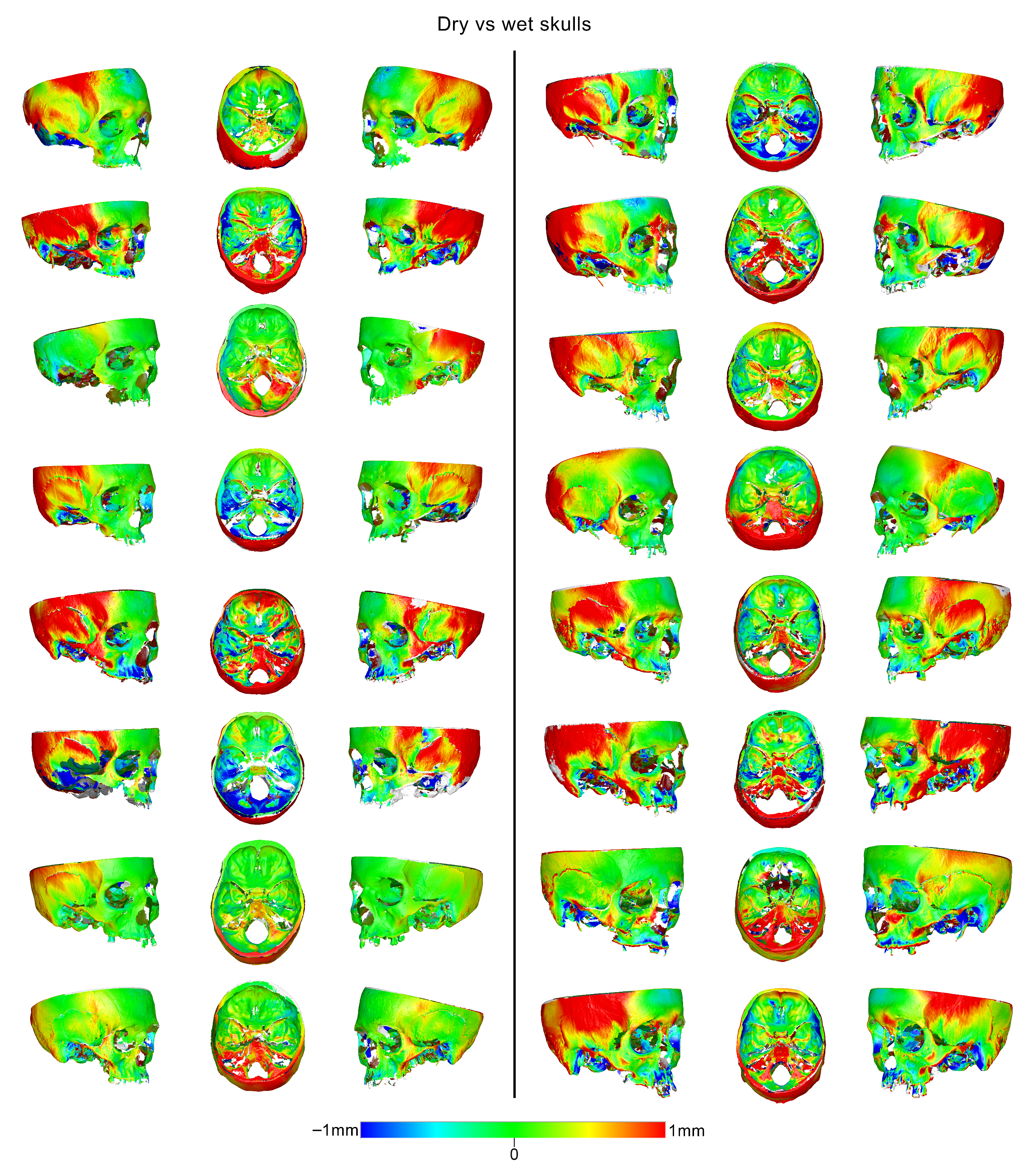


**Figure S1.** Color coded distance maps of best-fit superimpositions between 16 skulls scanned before (dry, set as reference model) and after hydration (through embedding in water for 15 min). Three skull views in a row per side correspond to a single specimen.


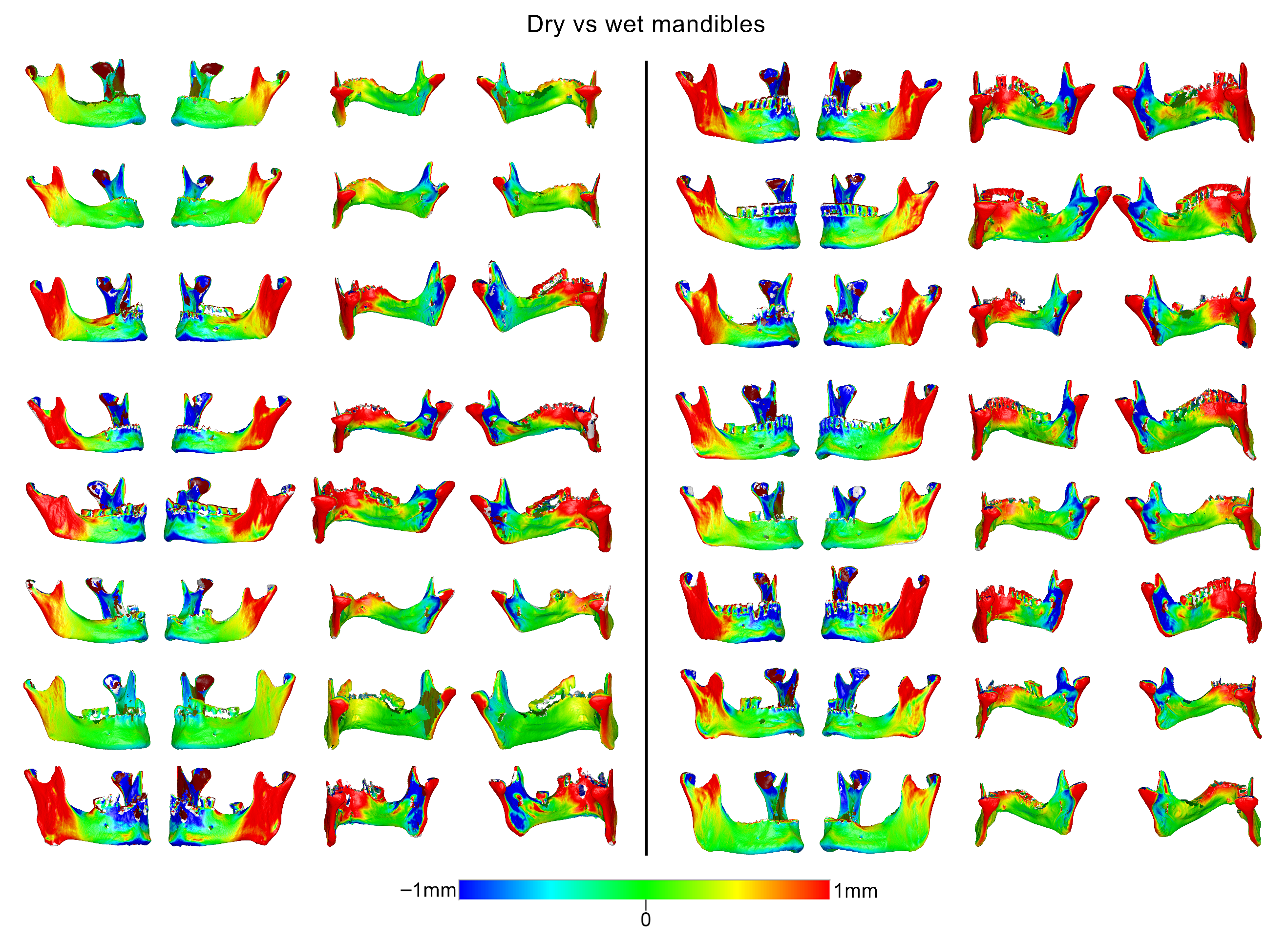


**Figure S2.** Color coded distance maps of best-fit superimpositions between 16 mandibles scanned before (dry, set as reference model) and hydration (through embedding in water for 10 min). Four mandibular views in a row per side correspond to a single specimen.


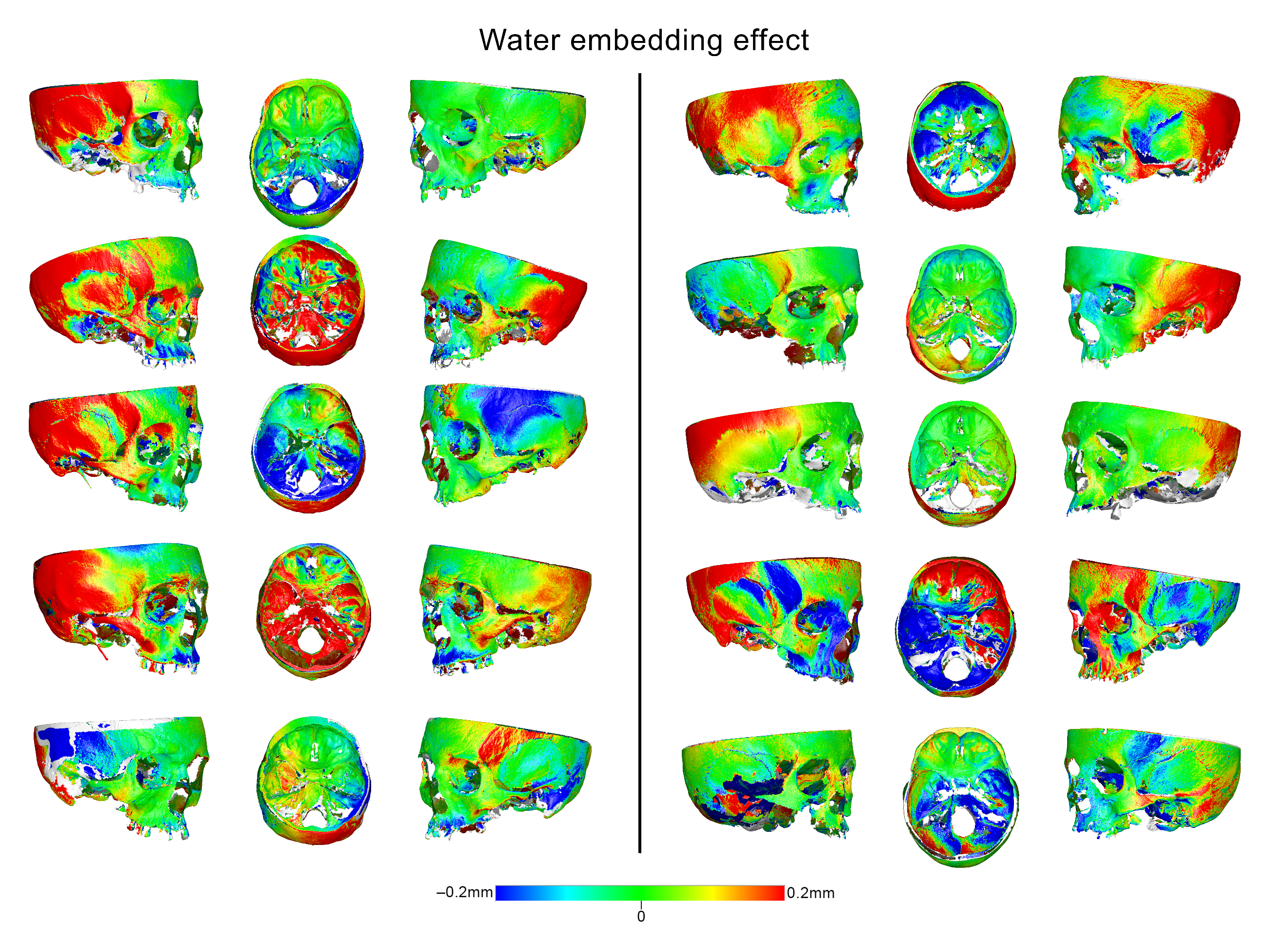


**Figure S3.** Color coded distance maps of best-fit superimpositions of 10 skulls embedded in water for different time periods (5 min, set as reference and 15 min). Three skull views in a row per side correspond to a single specimen.


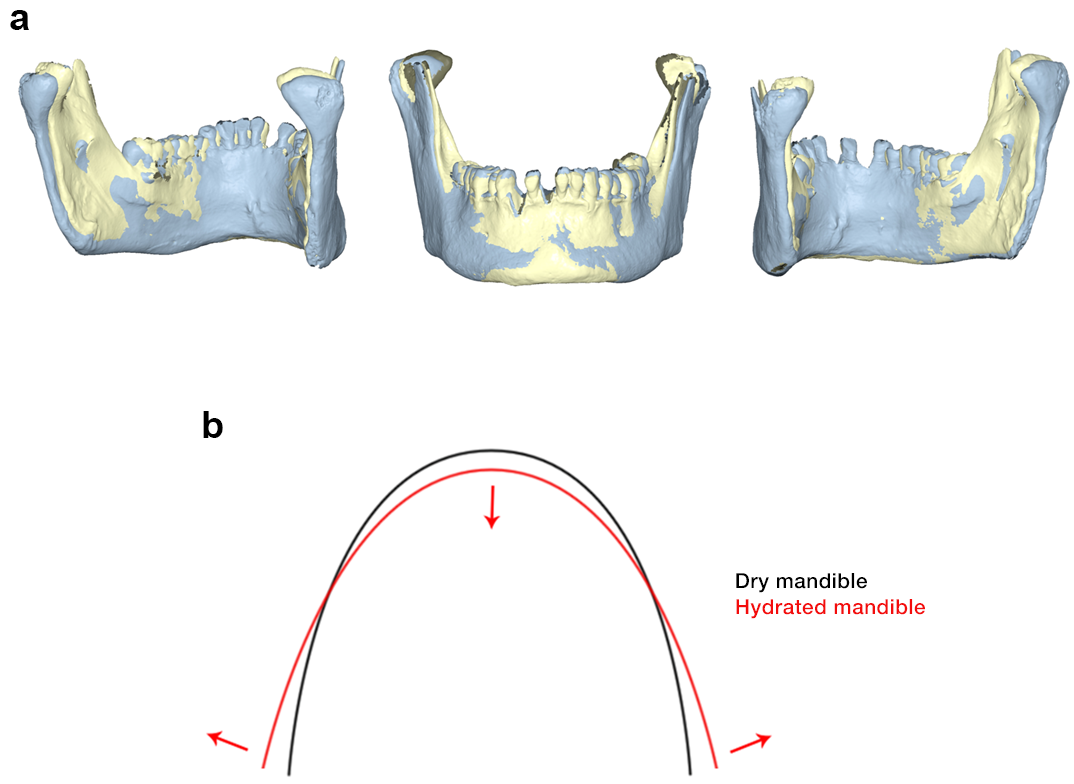


**Figure S4.** (**a**) Best fit superimposition of a mandibular specimen in dry (light yellow) and hydrated condition (light blue), using the same settings as described in the manuscript**.** The result demonstrates the flattening of the dry mandibles following hydration through embedding in water. (**b**) The effect is shown schematically in the drawing, where the dry condition is represented by the black line and the hydrated condition by the red line.


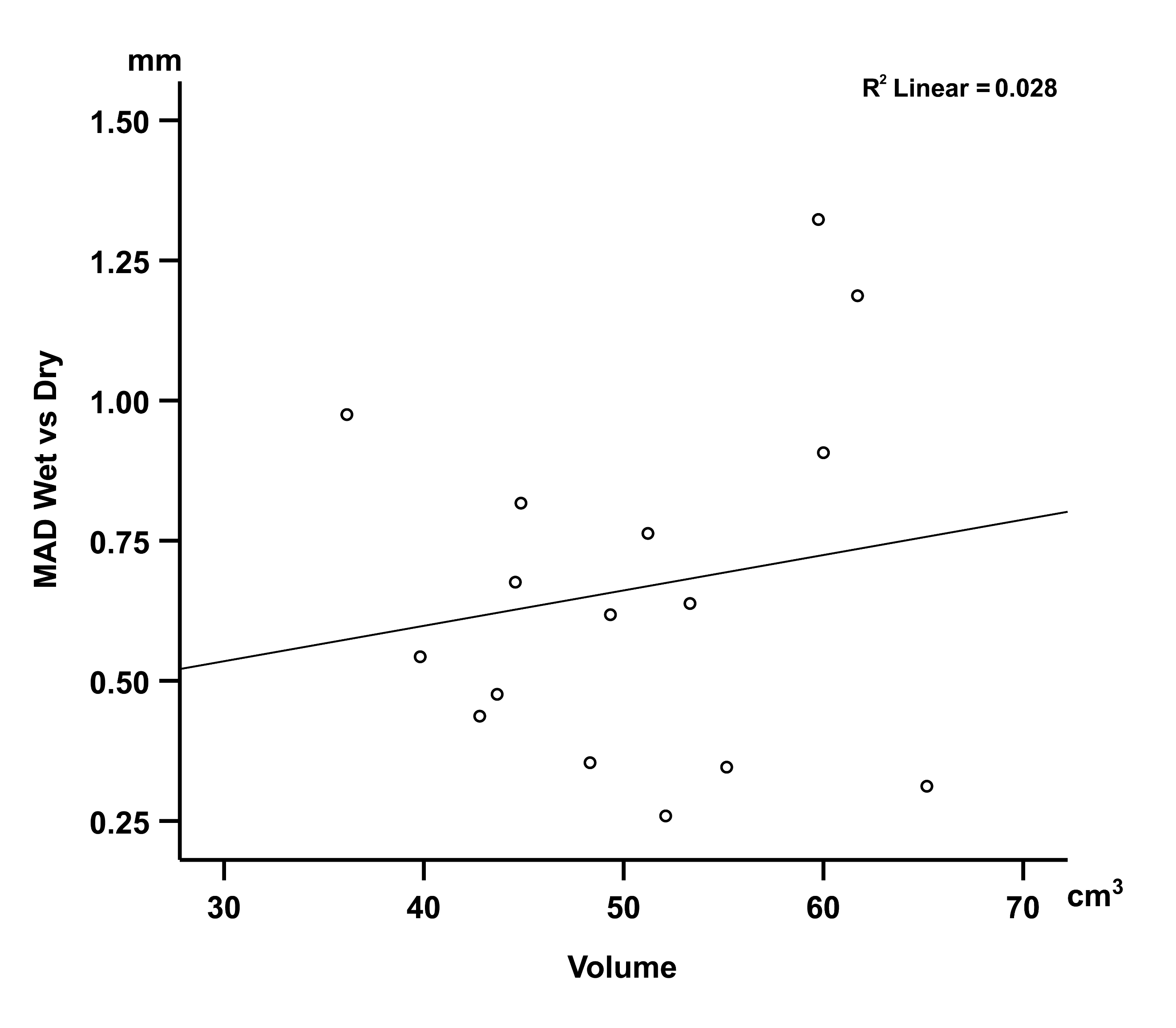


**Figure S5.** Scatter plot investigating the correlation between the initial volume of the dry mandibles and the detected mean absolute distance (MAD) of the dry from the corresponding wet model (hydration effect) on the six measurement areas tested. The linear regression did not detect any significant correlation.
